# Household

**Published:** 1887-05

**Authors:** 


### HOUSEHOLD.

INDIAN MUFFINS.— Two cups of corn meal; one cup of flour; one large tablespoonful of butter mixed with the corn meal; put enough milk in to make a batter; one egg; half cup light brown sugar ; two teaspoonfuls of royal baking powder mixed with salt and flour. Bake in quick oven.

STEWED BROWN BEANS.—Soak over night; put them in cold water and boil until soft; season them with molasses and vinegar according to taste ; thicken with flour or corn starch. Serve hot.

MUFFINS.—One quart sifted flour ; little salt ; one egg ; five teaspoonfuls baking powder; mix flour, salt, powder well together; add the egg with about one pint of milk ; mix as thick as cake.

PEA SOUP.—Soak the peas over night ; put them in cold water and let them boil ; keep skimming ; as they boil add cold water ; boil a little piece of salt pork with them ; season with an onion, cut fine ; add a little thyme. Strain and serve.

LEMON PIE.—Take the juice and rind of three good-sized lemons; stir in two tablespoonfuls of powdered sugar in the juice ; put a kettle with three cups of water over the fire, when it boils stir in two large tablespoonfuls of corn starch, mixed with cold water ; a small piece of butter and the beaten yolks of three eggs ; half-cup of white sugar; stir all together in kettle, let boil about a minute, then have the juice ready in a bowl and stir in the boiling mixture ; if not sweet enough add more sugar. Have two large-sized pie plates ready with plain crust, fill them up with mixture, place in hot oven and bake for one half hour.

FROSTING.—Take the whites of the eggs, with powdered sugar and beat to a froth. When pies are done frost them.

GERMAN CAKES.—One egg, seven ounces of butter, four ounces of powdered sugar, ten and one half ounces of flour, one tablespoonful of molasses. Mix without the wetting, and roll out; sprinkle cinnamon and sugar on top, roll again thinner, and roll out into little cakes.

LUNCH CAKES.—One cup of green corn pulp, one tablespoonful of sugar and enough fine oatmeal to make the mixture sufficiently stiff to drop it in spoonfuls on the pan. Bake for fifteen minutes and serve cold.

COCOANUT PUDDING.—Soak three tablespoonfuls tapioca in cold water over night, boil one quart of milk, add the tapioca and boil five minutes, then add the yolks of four eggs, four tablespoonfuls of sugar, three tablespoonfuls of dessicated cocoanut; boil ten minutes; turn into a dish to cool; beat the whites and two tablespoonfuls of sugar to a foam. Spread over the top and sprinkle over a little more cocoanut. Set in the oven to brown slightly.

MACARONI A I'ITALIENNE.—Drop half a pound of the smallest size into salted boiling water. It will be tender in from thirty to forty minutes. Drain and put on a hot dish containing two ounces of butter; sprinkle over three ounces of grated cheese; take a silver fork in each hand and toss the macaroni until the butter and cheese are melted. Season with salt and pepper and pour over it a few tablespoonsful of any good beef gravy and a cup of tomato sauce.

CURRIED RICE.—Wash a tablespoonful of rice, and boil in salted water rapidly until the grains burst. Do not stir with a spoon while cooking, but shake frequently to prevent sticking. Drain in a sieve; put in a hot dish and pour over it a third of a cup of butter, in which a tablespoonful of curry powder has been mixed.

PICKLED CHICKEN.—Boil four chickens until tender enough for meat to fall from the bones; put meat in a stone jar and pour over it three pints of cold, good cider vinegar and a half of the water in which the chickens were boiled: add spices if preferred, and it will be ready for use in two or three days. This is a popular Sunday evening dish; it is good for luncheon at any time.

EGG BISCUITS.—One quart of prepared flour, a tablespoonful of lard and twice as much butter, a teaspoonful of salt, two cups of milk, the yolks of two eggs, beaten light. Salt the flour and sift it twice in a bowl, rub in the shortening thoroughly and lightly; mix yolks and milk together, and pour into a hole in the flour; work into paste with as little handling as possible; roll into a sheet half an inch thick, cut into round cakes and bake in a floured pan. Eat hot.

